# Magnetoencephalographic study on facial movements

**DOI:** 10.3389/fnhum.2014.00550

**Published:** 2014-07-29

**Authors:** Kensaku Miki, Ryusuke Kakigi

**Affiliations:** ^1^Department of Integrative Physiology, National Institute for Physiological SciencesOkazaki, Japan; ^2^Department of Physiological Sciences, School of Life Science, The Graduate University for Advanced Studies (SOKENDAI)Hayama, Kanagawa, Japan

**Keywords:** MEG, facial movements, MT/V5, fusiform area, occipitotemporal area

## Abstract

In this review, we introduced our three studies that focused on facial movements. In the first study, we examined the temporal characteristics of neural responses elicited by viewing mouth movements, and assessed differences between the responses to mouth opening and closing movements and an averting eyes condition. Our results showed that the occipitotemporal area, the human MT/V5 homologue, was active in the perception of both mouth and eye motions. Viewing mouth and eye movements did not elicit significantly different activity in the occipitotemporal area, which indicated that perception of the movement of facial parts may be processed in the same manner, and this is different from motion in general. In the second study, we investigated whether early activity in the occipitotemporal region evoked by eye movements was influenced by the facial contour and/or features such as the mouth. Our results revealed specific information processing for eye movements in the occipitotemporal region, and this activity was significantly influenced by whether movements appeared with the facial contour and/or features, in other words, whether the eyes moved, even if the movement itself was the same. In the third study, we examined the effects of inverting the facial contour (hair and chin) and features (eyes, nose, and mouth) on processing for static and dynamic face perception. Our results showed the following: (1) In static face perception, activity in the right fusiform area was affected more by the inversion of features while that in the left fusiform area was affected more by a disruption in the spatial relationship between the contour and features; and (2) In dynamic face perception, activity in the right occipitotemporal area was affected by the inversion of the facial contour.

## Introduction

The “Face” provides much important information in our daily lives. Many studies on face perception that used microelectrodes on monkeys and humans detected face-specific neurons in the temporal cortex, mainly in the superior temporal sulcus (STS), and convexity of the inferior temporal (IT) cortex. Face perception processes have been reported in psychological studies (e.g., Bruce and Young, 1986), and many studies have examined the mechanisms underlying human face perception in detail using neuroimaging methods, including electroencephalography (EEG) recorded from the scalp (event-related potentials, ERPs) and cortical surface (electrocorticography, ECoG), magnetoencephalography (MEG), functional magnetic resonance imaging (fMRI), and near infrared spectoroscopy (NIRS).

Two important factors for face perception are static face perception and facial movement perception. The fusiform gyrus in the IT area may be specific to static face perception. A negative ERP component, being maximum at approximately 170 ms, is evoked by face stimuli in the bilateral temporal area. This has been referred to as N170. N170 is known to be larger for the face than for other objects (for example, a car and chair), and this reflects face perception (e.g., Rossion and Jacques, 2008). In ECoG studies, a negative component (N200) was detected in the small regions in the fusiform and IT gyrus evoked by faces but not by other stimuli (Allison et al., 1994a,b). The ECoG can been used to investigate the temporal and spatial aspects of the mechanisms responsible for face in detail, but it is an invasive method. MEG also has high temporal and spatial resolutions, and it is a non-invasive method. Therefore, MEG is useful to investigate face perception in normal volunteers. In MEG studies, a component was found to be evoked by the face, M170, which corresponded to N170 in EEG studies, and its activity was estimated to be in the fusiform area (e.g., Watanabe et al., 1999; Halgren et al., 2000). In fMRI studies with a very high spatial resolution, the fusiform face area (FFA) was identified in the fusiform gyrus and is selectively involved in the perception of faces (Kanwisher et al., 1997).

Recognizing facial movements is very important in addition to recognizing the static face, for example, in social communication and non-language communication. In monkeys, a neuronal population in the anterior superior temporal polysensory area (STPa) responded selectively to the motion of animate objects, including bodies and faces (e.g., Oram and Perrett, 1994). In recent monkey studies using fMRI, a different pattern was observed in the anterior STS, which responded more to dynamic than static faces, but was not sensitive to dot motion (Furl et al., 2012).

Many human studies have been conducted on static face perception, whereas a smaller number of studies have investigated facial movement perception using non-invasive neuroimaging methods. In fMRI studies, the STS in addition to MT/V5, which is considered to play an important role in motion perception, was activated when facial movement was viewed (Puce et al., 1998; Schultz and Pilz, 2009). In ERP studies (Wheaton et al., 2001; Thompson et al., 2002; Puce and Perrett, 2003; Puce et al., 2003), the amplitude of N170 in the occipitotemporal area evoked by facial (mouth) movement with a line-drawn face was larger than that evoked by general motion with a spatially “scrambled” line-drawn face. In a recent event-related spectral perturbations (ERSPs) study, occipitotemporal beta and gamma activities differentiated between facial and non-facial motion (Rossi et al., 2014). In our previous MEG studies (Watanabe et al., 2001; Miki et al., 2004, 2007), the activities by eyes and mouth movements of the occipitotemporal area, corresponding to human MT/V5, was different from movements in general. Consistent with our findings, a recent MEG study reported that cortical responses to eye blinks were clearly differently than expected on the basis of simple physical characteristics (Mandel et al., 2014). Moreover, Ulloa et al. (2014) reported that the initial gaze change elicited a significantly larger M170 under the deviated than mutual attention scenario.

Based on previous studies, we determined (1) whether processing of the perception of facial movements was specific and different from motion in general; (2) what information within the face was important to the processing of facial movement perception if this processing was specific; and (3) whether the right hemisphere played a more important role in facial movement perception than the left. Since we previously studied brain activities evoked by viewing various types of human facial movements based on our hypothesis, we herein introduced three representative studies: (a) Magnetoencephalographic study of occipitotemporal activity elicited by viewing mouth movements (Miki et al., 2004); (b) Effects of face contour and features on early occipitotemporal activity when viewing eye movement (Miki et al., 2007); and (c) Effects of inverting contour and features on processing for static and dynamic face perception: an MEG study (Miki et al., 2011).

## Magnetoencephalographic study of occipitotemporal activity elicited by viewing mouth movements

Facial movements are useful for social communication in humans. For example, the direction of the eye gaze is used to assess the social attention of others, and moreover, it becomes markedly easier to understand speech when we can see the mouth movements of the speaker. In a previous MEG study (Watanabe et al., 2001), a specific region for the perception of eye movements was detected within the occipitotemporal area, corresponding to human MT/V5, and this was different from motion in general. We hypothesized that the perception of the movement of facial parts may also be processed in a similar manner, which is different from motion in general. Therefore, the main objectives of the first study were to examine the temporal characteristics of the brain activity elicited by viewing mouth movements (opening and closing), and compare them to those of eye aversion movements and motion in general.

**Figure 1:**
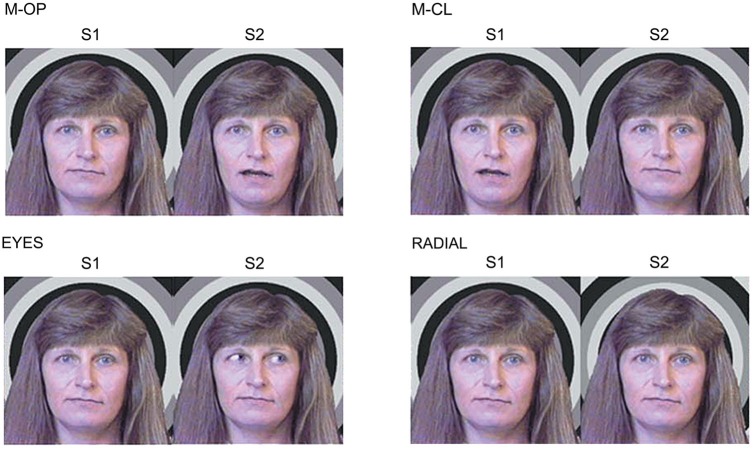
**Examples of the stimulus conditions**. (1) M-OP: the mouth is opening, (2) M-CL: the mouth is closing, (3) EYES: the eyes are averted, and (4) RADIAL: a radial stimulus moving inward (adopted from Miki et al., 2004).

Seventeen right-handed adults (4 females, 13 males: 24–43 (mean age 32.2) years) with normal or corrected visual acuity participated in this study. We used apparent motion, in which the first stimulus (S1) was replaced by a second stimulus (S2) with no inter-stimulus interval as follows (Figure 1):

1. M-OP: The mouth is opening.
2. M-CL: The mouth is closing.
3. EYES: The eyes are averted.
4. RADIAL: A radial stimulus moving inward.

**Figure 2:**
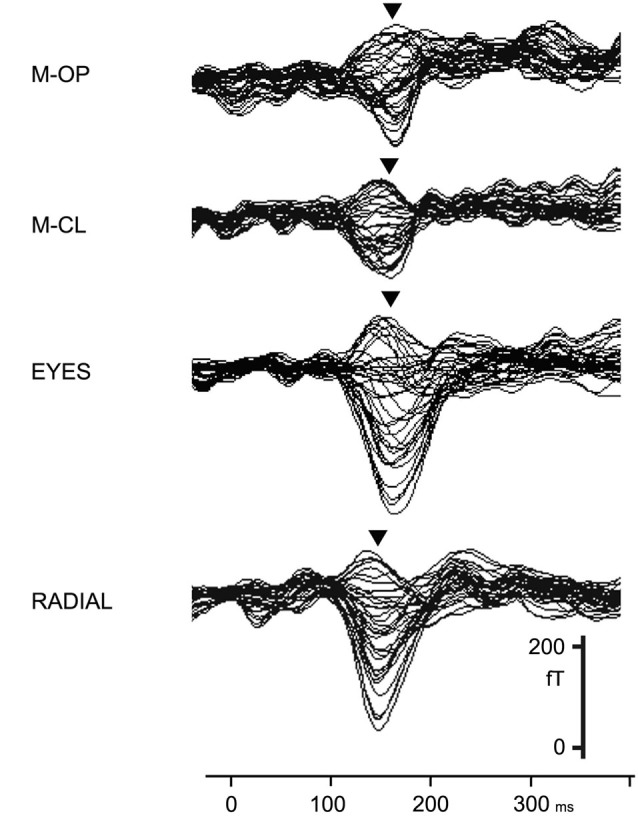
**Right hemisphere MEG activity shown in a 37-channel superimposed display for all conditions in a representative subject (adopted from Miki et al., 2004)**.

A large clear component, 1M, was elicited by all conditions (M-OP, M-CL, EYES, and RADIAL) within 200 ms of the stimulus onset (Figure 2). Concerning the peak latency of 1M, the means and standard deviations were 159.8 ± 17.3, 161.9 ± 15.0, 161.2 ± 18.9, and 140.1 ± 18.0 ms for M-OP, M-CL, EYES and RADIAL in the right hemisphere, respectively, and 162.4 ± 11.6, 160.9 ± 9.8, 164.6 ± 14.2, and 138.4 ± 9.0 ms for M-OP, M-CL, EYES, and RADIAL in the left, respectively. The latency for RADIAL was significantly shorter than that for the facial motion conditions (*p* < 0.05). No significant differences were observed in 1M latency between M-OP, M-CL, or EYES.

We used a multi-dipole model, brain electric source analysis (BESA; Scherg and Buchner, 1993) (Neuroscan, McLean, VA) computation of theoretical source generators in a three-layer spherical head model, and estimated activity in the occipitotemporal area, the human MT/V5 area homologue, from 1M. The means and standard deviations of the dipole moment of the estimated dipoles from 1M was 7.9 ± 1.9, 7.8 ± 3.2, 10.0 ± 6.8, and 13.8 ± 4.9 nAm for M-OP, M-CL, EYES, and RADIAL in the right hemisphere, respectively, and 7.4 ± 2.8, 6.7 ± 3.0, 9.3 ± 4.3, and 13.6 ± 1.8 nAm for M-OP, M-CL, EYES, and RADIAL in the left, respectively. No significant differences were observed in the dipole moment (strength) for M-OP, M-CL and EYES between either hemisphere. However, M-OP and M-CL were significantly smaller than RADIAL (*p* < 0.05) in the right hemisphere and M-OP, M-CL, and EYES were significantly smaller than RADIAL (*p* < 0.05) in the left.

The results of the first study indicated that the occipitotemporal (human MT/V5) area was active in the perception of both mouth and eye movement. Furthermore, viewing mouth and eye movements did not elicit significantly different activity in the occipitotemporal (human MT/V5) area, which suggested that the perception of the movement of facial parts may be processed in the same manner, and this is different from motion in general.

## Effects of face contour and features on early occipitotemporal activity when viewing eye movement

The first study showed that the perception of the movement of facial parts may be processed in the same manner, and this is different from motion in general. However, the main factor(s) causing differences in recognizing facial versus general movement have yet to be elucidated in detail.

Many studies have investigated effect facial contour and features using a static face. A previous study reported that it took longer to recognize an eyes-only stimulus or only facial features (eyes, nose, and mouth) than a full-face stimulus with a contour (e.g., Watanabe et al., 1999), and the contour of the face is important in static face recognition. However, to the best of our knowledge, the effects of the facial contour and features on facial movement recognition have not yet been investigated. Therefore, the main objectives of the second study were to investigate the effects of facial contour and features on early occipitotemporal activity evoked by facial movement. We also used a schematic face because a simple schematic drawing with a circle for a contour, two dots for eyes, and a straight line for lips, was recognized as a face even though each individual component of the drawing by itself was not. Previous studies using a schematic face showed that N170 was evoked by schematic faces as well as photographs of real faces (Sagiv and Bentin, 2001; Latinus and Taylor, 2006).

**Figure 3:**
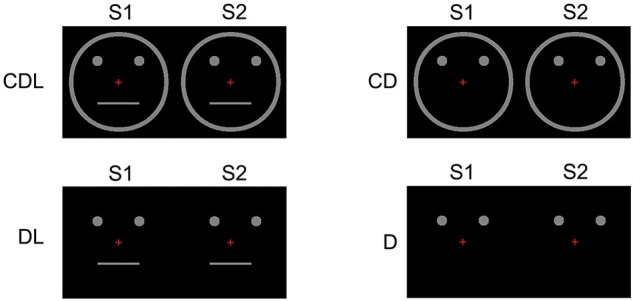
**Examples of the stimulus conditions.** (1) CDL: schematic face consisting of a Contour, two Dots, and a horizontal Line, (2) CD: the Contour and two Dots, (3) DL: two Dots and a horizontal Line, and (4) D: two Dots only (adopted from Miki et al., 2007).

Thirteen right-handed adults (6 females, 7 males: 24–46 (means; 33.6) years) with normal or corrected visual acuity participated in this study. We used apparent motion and presented the following four conditions (Figure 3):

1. CDL: A schematic face consisting of a facial Contour, two Dots, and a horizontal Line.
2. CD: The Contour and two Dots.
3. DL: Two Dots and a horizontal Line.
4. D: Two Dots only.

Subjects described the simple movement of dots for D, whereas eye movement for CDL, CD, and DL, though movement modalities were the same for all conditions. In source modeling, we used a single equivalent current dipole (ECD) model (Hämäläinen et al., 1993) within 145–220 ms of the stimulus onset.

**Figure 4:**
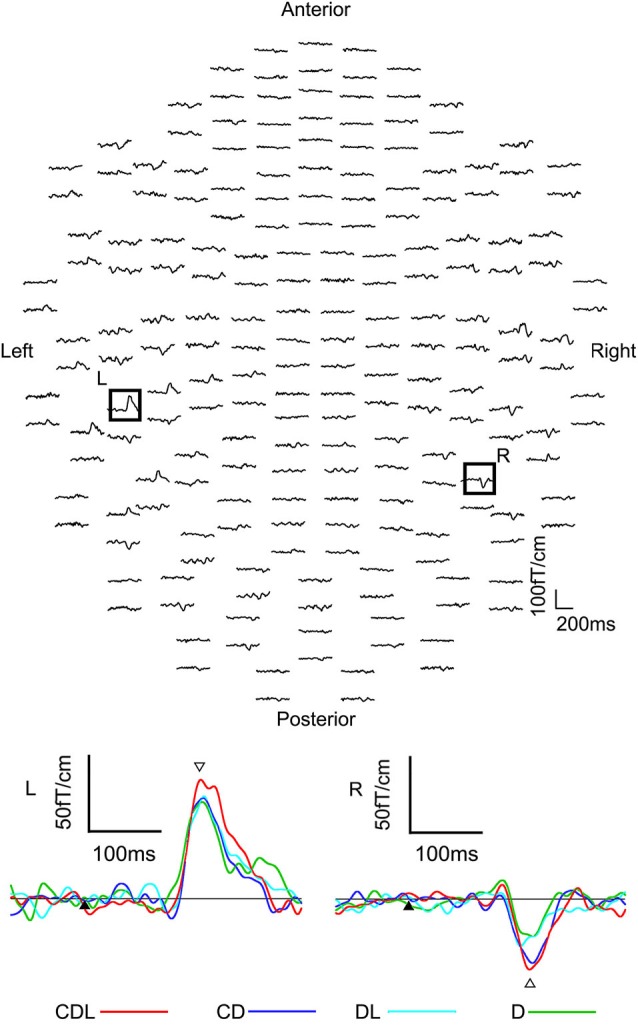
**The waveforms recorded from 204 gradiometers of a representative subject following the S2 onset (eye movements) in the CDL condition.** R: representative waveforms at sensor R in the right hemisphere of the upper image. L: representative waveforms at sensor L in the left of the upper image. Black arrows are the S2 onset. White arrows indicates the main response after the S2 onset (adopted from Miki et al., 2007).

Clear MEG responses were elicited in all conditions (CDL, CD, DL, and D) at the sensors in the bilateral occipitotemporal area (Figure 4). The means and standard deviations of the peak latency of the estimated dipole was 179.3 ± 26.3, 183.0 ± 16.9, 180.9 ± 20.8, and 180.3 ± 23.7 ms for CDL, CD, DL, and D in the right hemisphere, respectively, and 180.2 ± 14.9, 180.5 ± 24.8, 174.0 ± 24.9, and 177.7 ± 20.3 ms for CDL, CD, DL, and D in the left, respectively. No significant differences were observed among any condition.

The means and standard deviations of the dipole moment was 14.4 ± 6.2, 11.2 ± 7.9, 9.6 ± 6.5, and 10.3 ± 5.3 nAm for CDL, CD, DL, and D in the right hemisphere, respectively, and 12.7 ± 6.7, 11.1 ± 6.1, 9.6 ± 5.4, and 8.9 ± 5.5 nAm for CDL, CD, DL, and D in the left, respectively. The moment was significantly larger for CDL than for CD (*p* < 0.05), DL (*p* < 0.01), and D(*p* < 0.01) in the right hemisphere, and for CDL than for DL and D (*p* < 0.01) in the left hemisphere.

Our results in the second study demonstrated specific information processing for eye movements, which was different from motion in general, and activity in the occipitotemporal (human MT/V5) area related to this processing was influenced by whether movements appeared with the contour and/or features of the face.

## Effects of inverting contour and features on processing for static and dynamic face perception: an MEG study

The second study showed that the activity evoked in the occipitotemporal area by eye movements was influenced by the existence of the contour and/or features of the face. However, it remained unclear whether this activity was influenced by the orientation of the contour and/or features of the face.

In static face perception, the N170 component was longer and larger for an inverted face than for upright face (Bentin et al., 1996; Sagiv and Bentin, 2001; Itier and Taylor, 2004; Latinus and Taylor, 2006), which indicated that N170 was affected by inversion of the face, i.e., the face inversion effect. In addition, the latency of N170 was longer for scrambled features than for upright faces (George et al., 1996; Latinus and Taylor, 2006), which confirmed that N170 was affected by a disruption in the spatial relation between the facial contour and features.

Based on the findings of previous N170 studies on static face perception, we hypothesized that the perception of eye movements may mainly be affected by information on the contour and other facial features. Therefore, the main objectives of the third study were to investigate the effects of inverting the facial contour and features on the occipitotemporal (human MT/V5) area related to a dynamic face and what information within the face was important for processing dynamic face perception. We also investigated the effects of inverting the facial contour and features of the face on the fusiform area related static face perception to compare with the occipitotemporal area.

**Figure 5:**
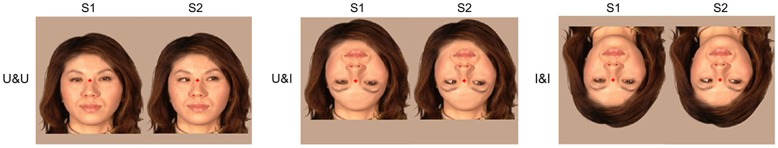
**Examples of the stimulus conditions**. (1) U&U: upright contour (hair and chin) and Upright features (eyes, nose, and mouth), (2) U&I: upright contour and Inverted features, and (3) I&I: inverted contour and Inverted features (adopted from Miki et al., 2011).

We recruited 10 right-handed adults (3 females and 7 males: 24–47 (means; 30.6) years) with normal or corrected visual acuity. We used apparent motion and presented the following three conditions (Figure 5):

1. U&U: Upright contour and Upright features.
2. U&I: Upright contour and Inverted features. In the U&I condition, the spatial relationship between the facial contour and features was disrupted, and this was different from U&U condition.
3. I&I: Inverted contour and Inverted features. In the I&I condition, the spatial relationship between the facial contour and features was unchanged, which was also the case for the U&U condition.

The eyes were averted to the right of the viewer under all conditions.

As in the second study, we used a single ECD model (Hämäläinen et al., 1993) and estimated the dipole within 105–200 ms of the S1 onset (static face) and 115–210 ms of the S2 onset (eye movements).

In static face perception (S1 onset), ECDs were estimated to lie in the fusiform area from MEG following S1 in all conditions.

**Figure 6:**
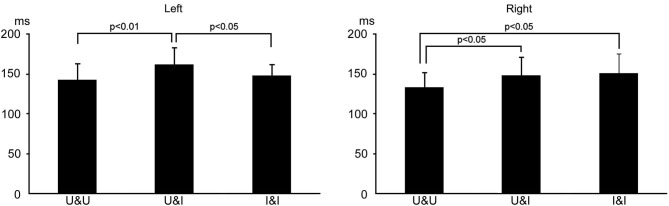
**Bar graphs of peak latency for all conditions after S1 onset in the right and left hemispheres**. Error bars show the standard deviation (S.D.).

The means and standard deviations of the peak latency in activity in the fusiform area was 133.6 ± 18.5, 148.4 ± 22.4, and 151.4 ± 24.1 ms for U&U, U&I, and I&I in the right hemisphere, respectively, and 143.2 ± 19.7, 162.2 ± 21.0, and 148.4 ± 13.6 ms for U&U, U&I, and I&I in the left, respectively. Latency was significantly longer for U&I (Upright contour and Inverted features) (*p* < 0.05) and I&I (Inverted contour and Inverted features) (*p* < 0.05) than for U&U in the right hemisphere, and also for U&I than for U&U (*p* < 0.01) and I&I (*p* < 0.05) in the left (Figure 6).

The means and standard deviations of the strength (the maximum of the dipole moment) of activity in the fusiform area was 28.6 ± 21.1, 35.5 ± 18.5, and 34.4 ± 18.5 nAm for U&U, U&I, and I&I in the right hemisphere, respectively, and 21.9 ± 13.5, 20.3 ± 11.8, and 21.9 ± 14.5 nAm for U&U, U&I, and I&I in the left, respectively. No significant differences were observed in the maximum of the dipole moment among the three conditions.

In dynamic face perception (S2 onset), ECDs were estimated to lie in the occipitotemporal area, the human MT/V5 area homologue, from MEG following S2 in all conditions.

The means and standard deviations of the peak latency of activity in the occipitotemporal area was 163.8 ± 31.3, 159.4 ± 22.3, and 159.7 ± 25.1 ms for U&U, U&I, and I&I in the right hemisphere, respectively, and 151.6 ± 22.1, 157.8 ± 24.4, and 151.7 ± 23.8 ms for U&U, U&I, and I&I in the left, respectively. No significant differences were observed in the peak latency among the three conditions.

**Figure 7:**
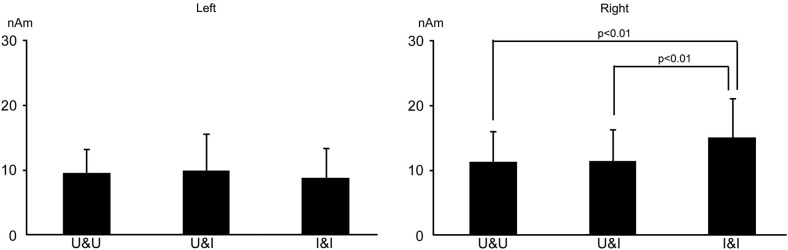
**Bar graphs of dipole moment for all conditions after S2 onset in the right and left hemispheres**. Error bars show the standard deviation (S.D.).

The means and standard deviations of the strength (the maximum of the dipole moment) of activity in the occipitotemporal area was 11.3 ± 4.6, 11.5 ± 4.8, and 15.1 ± 5.9 nAm for U&U, U&I, and I&I in the right hemisphere, respectively, and 9.6 ± 3.7, 10.0 ± 5.6, and 8.8 ± 4.6 nAm for U&U, U&I, and I&I in the left, respectively. The maximum of the dipole moment was larger for I&I than for U&U and U&I in the right hemisphere (*p* < 0.01), but not the left (Figure 7).

The results of the third study indicated the following: (a) considering the fusiform area related to static face perception, activity was affected more by the inversion of features in the right hemisphere while it was affected more by a disruption in the spatial relationship between the facial contour and features in the left hemisphere; and (b) considering the occipitotemporal (human MT/V5) area related to dynamic face perception, activity was affected by the inversion of the facial contour in the right, but not in the left hemisphere.

## Summary and conclusion

In our three studies, we focused on activity in the occipitotemporal area, the human MT/V5 homologue, related to facial parts movement. We summarized our results as followings: (1) viewing mouth and eye movements did not elicit significantly different activity in the occipitotemporal area; (2) neuronal activities in the occipitotemporal area evoked by facial (eye) movements were affected by whether the contour and/or features of the face were in the stimulus; (3) the activity of the right occipitotemporal area evoked by facial (eye) movements was affected by the inversion of the facial contour, and these results indicated the following: (1) processing of the perception on facial movements is specific and is different from motion in general; (2) the existence of the facial contour and face parts are important factors in the perception of facial movements; (3) the orientation of the contour and spatial relationship between the contour and facial parts are also important; and (4) the right occiptiotemporal area is more important in the perception of the facial movements than the left. Based on the results of the three experiments, it still remains unclear how the transmission of facial movement processing was modulated by facial form information.

Connectivity models that modeled communication between the ventral form and dorsal motion pathways were tested in a recent fMRI study related to perception of dynamic faces (Furl et al., 2014), and the findings obtained clearly showed that facial form information modulated the transmission of motion from V5 to the STS. Based on these findings, we hypothesized that information on the facial contour and parts, transmitting via FFA and/or OFA (occipital face area), may gate the transmission of information regarding facial motion via MT/V5, and that facial form and motion information may have been integrated in the STS. We consider these results and hypothesis to be useful for investigating the functional roles of human brain connectomes and also provide an insight into facial movement processes.

## Conflict of interest statement

The authors declare that the research was conducted in the absence of any commercial or financial relationships that could be construed as a potential conflict of interest.
